# Emotional feedback ameliorates older adults’ feedback-induced learning

**DOI:** 10.1371/journal.pone.0231964

**Published:** 2020-04-30

**Authors:** Nicola K. Ferdinand, Melanie Hilz

**Affiliations:** 1 Department of Psychology, Bergische Universität Wuppertal, Wuppertal, Germany; 2 Department of Psychology, Saarland University, Saarbrücken, Germany; Texas A&M University, UNITED STATES

## Abstract

In older age, learning and feedback processing are usually impaired. This is thought to be due to impairments in the dopaminergic system and the anterior cingulate cortex. By contrast, processing of affective information seems to remain relatively intact. Recent research has also demonstrated that cognitive functioning can be influenced by affective materials or contexts and lead to an enhancement in diverse cognitive tasks. Hence, the aim of the present study was to explore, whether emotional feedback would counteract age-related learning deficits and strengthen early and later phases of feedback processing as reflected in the feedback-related negativity (FRN) and P3b of the event-related potential (ERP). Younger and older participants conducted a probabilistic reinforcement learning task in which the accurate responses had to be learned via feedback. In emotional trials, feedback stimuli consisted of faces with smiling and disgusted expressions, and in a non-emotional condition, positive and negative feedback was indicated by the background color of faces with neutral expressions. Our main results were that older adults showed better learning performance in the emotional feedback condition and a larger P3b after emotional than non-emotional feedback indexing heightened working memory updating after task relevant events.

## Introduction

In order to learn which behavior leads to a desired goal humans need to be able to process feedback from their environment. In an ever-changing world, this ability is still very important in old age. Many studies have shown that older adults have impairments in feedback-induced learning which is probably due to deficits in feedback processing [e.g., 1–2]. By contrast, processing of affective information seems to remain relatively intact [e.g., 3]. It has also been demonstrated that cognitive functioning can be influenced by affective information or affective contexts and that this can lead to an enhancement in diverse cognitive tasks [e.g., 4–5]. Thus, the goal of the present study was to explore whether emotional feedback can ameliorate age-related impairments in feedback-induced learning.

It is well known that processing and learning from feedback critically depends on the integrity of the mediofrontal cortex and the mesencephalic dopamine system. While mesencephalic dopamine neurons signal unpredicted rewards and punishments [6–8], the mediofrontal cortex, more specifically the anterior cingulate cortex (ACC), uses these reward prediction errors as reinforcement learning signals to successfully adapt the behavior to the present tasks and goals [e.g., 7, 9].

Using event-related potentials (ERPs), it has been found that feedback is processed in several phases [cf. 10]. In a fast initial phase which is reflected in the feedback-related negativity (FRN). The FRN, which is probably generated in the ACC, is measured over fronto-central scalp regions after participants have received feedback [e.g., 7, 11–14]. It reflects the detection of a reward prediction error, e.g., unexpected negative feedback [e.g., 12–13, 15–16]. In the recent literature, the FRN has been measured in different ways. When a peak-to-peak amplitude measure is used, this indexes the size of the reward prediction error, i.e., the expectancy violation [e.g., 11]. It can also be measured by a mean amplitude measure. In this case, a larger negativity is usually found after negative than positive feedback [e.g., 7]. In addition, a later and slower processing step is reflected in the P3b after the feedback stimulus. This component has been linked to updating processes in working memory that take place after an unexpected or surprising event [cf. 17–18]. It is also related to the task relevance of these unexpected events [e.g., 19–20].

Older adults usually show attenuated feedback processing as reflected in a reduced FRN [21–27] and in a reduced ability to learning from feedback [28,29,1, 2]. It has been argued that these age-related impairments result from a weakened dopaminergic reinforcement learning signal [30–33]. These age-related impairments in dopamine system have also been linked to older adults’ working memory problems [for a review, see 31]. This fits empirical results, demonstrating differences in amplitude and topography of the P3b between younger and older adults during feedback processing [e.g., 10, 34]. Hence, in old age all the above steps of feedback processing appear to be attenuated.

On the other hand, there is ample evidence that processing of emotional information does not decline in older age. In line with this idea, it has been shown that while cognitive prefrontal circuits are strongly affected in old age, this is not the case for affective ones [e.g., 3]. A number of studies have also found that emotional influences can enhance performance in diverse cognitive tasks [e.g., 4] and also improve memory performance or attention in older adults [35–38]. The socio-emotional selectivity theory has explained this heightened processing of emotional content by stating that when future time horizon are perceived as limited, older adults start to focus on emotional well-being [35, 38]. Therefore, using emotional materials or contexts might be an adequate means to attenuate impairments in old age. But to what extent these beneficial effects of emotion generalize to other domains of cognitive functioning, like feedback processing and feedback-induced learning, is still an open issue.

Only a few studies have explicitly contrasted emotional and non-emotional types of feedback in the same paradigm and even less examined age differences therein. For younger adults, there seems to be emerging evidence that emotional feedback leads to enhanced feedback processing and better learning performance than abstract performance feedback. For example, Hurlemann and colleagues [39] compared abstract feedback (red light vs. green light) with emotional feedback (angry vs. smiling face) in a purely behavioral learning paradigm. Their results showed improved learning in conditions with emotional feedback. Similarly, Dekkers and colleagues [40] found heightened feedback processing as reflected in a larger feedback-locked P3 (but not FRN) in young female adults in an emotional social-judgment task as compared with a less emotional age-judgment task. Pfabigan, Gittenberger and Lamm [41] examined feedback processing in a time estimation task. They compared social hand gestures (thumps up vs. thumps down) as feedback stimuli with non-social feedback stimuli (plus vs. minus sign). Although they found no differences in task performance, they found that the FRN as well as the P3b was larger after social than non-social feedback stimuli [see also 42]. Taken together, emotional feedback seems to strengthen learning and feedback processing in younger adults, although there are hints that other types of rewarding feedback, like monetary rewards, are still more effective than affective feedback [43–44].

To our knowledge, although there is reason to believe that emotional feedback should be especially helpful for older adults, there are no similar electrophysiological studies comparing emotional and non-emotional feedback processing in older adults. However, there are two behavioral studies comparing cognitive feedback with emotional feedback in learning tasks. Gorlick and colleagues [45] investigated younger and older participants in a rule learning task including abstract cognitive (point loss vs. gain) or emotional (angry vs. happy face) feedback. For younger adults, no learning differences between the two conditions were found. Older adults, however, showed impairments in learning performance, which were attenuated by the emotional feedback. More specifically, in tasks with a low cognitive load, happy-face feedback reduced age-related deficits, while in tasks with a high cognitive load, angry-face feedback reduced age-related deficits. In contrast to Gorlick et al. [45], Nashiro, Mather, Gorlick, and Nga [46] did not find a benefit for emotional feedback in older adults. The authors compared younger and older participants’ performance on a reversal learning task. Participants had to learn via feedback in three different feedback conditions: a) a negative emotional condition with neutral faces as positive and angry faces as negative feedback, b) a positive emotional condition with happy faces as positive and neutral faces as negative feedback, and c) a non-emotional condition with “0 points” as negative and “+100 points” as positive feedback. It was found that older adults made more errors than younger adults in the negative emotional condition, but no age differences were present in the positive emotional condition.

The aforementioned studies examining feedback processing and learning suggest that the effect of different feedback types might vary with age and that affective feedback may be especially effective in older adults. Nevertheless, the results are mixed and studies comparing emotional and non-emotional feedback processing in younger and older adults by means of ERPs are lacking. However, using an ERP approach could be especially helpful to determine the underlying neuronal mechanisms and the corresponding cognitive processes that are modulated by emotion. Therefore, the goal of the present study was to explore whether emotional feedback in form of emotional faces can ameliorate age-related impairments in feedback processing and feedback-induced learning. For this reason, younger and older participants were invited to perform a probabilistic learning task in which the correct responses to a stimulus had to be inferred from via feedback. An emotional condition in which the feedback consisted of faces with smiling and disgusted expressions was compared to a non-emotional condition, in which positive and negative feedback was conveyed by the background color of faces with neutral expressions. During the task, behavioral responses as well as feedback-locked ERPs were recorded. Our hypotheses were that emotional feedback enhances feedback processing as reflected in a larger peak-to-peak FRN and P3b and that this effect should be especially pronounced for older adults. Better feedback processing in the emotional feedback condition should also reflect on learning performance and result in faster and better learning than as compared to the non-emotional feedback condition.

## Materials and methods

### Participants

A power analyses for the planned ERP analyses (see Section “Statistical Analyses” below) was done before the experiment was recorded and the sample size was determined accordingly: If a small to middle-sized effect size is assumed (f = .15) and an α of 0.05 together with a power of β = .8 is selected to examine the within-factors Feedback Condition (emotional, neutral), Feedback Valence (positive, negative), and Anterior/Posterior (Fz, Cz, Pz), (as repeated measures with an assumed correlation among repeated measures of .75), a power analysis indicates a minimal group size of 18 participants per group.

Thus, to compensate for possible drop-outs, the study examined 24 younger (19–29 years, 12 female/ 12 male) and 24 older adults (70–79 years, 12 female/ 12 male). They all reported good health and normal or corrected-to-normal vision. Forty-four of the 46 participants were right-handed, one younger participant was left-handed, and one older participant reported being a retrained left-hander. One younger participant had to be excluded from all subsequent analyses because his values in the behavioral data were more than three standard deviations away from the mean. In addition, one elderly participant was excluded from further analysis because of excessive artifacts in the EEG data. Consequently, the final sample consisted of 23 persons per age group (see Table 1). The study was conducted in agreement with the Declaration of Helsinki and the local ethics committee at Universität des Saarlandes endorsed the study design. Before the experiment started, all subjects signed informed consent. They were paid 24€ for their participation.

**Table 1:** Sample overview and results of psychometric tests (means and standard deviations).

	Younger adults	Older adults
n (female/ male)	23 (12/11)	23 (11/12)
Mean age (years) (*SD*)	22.78 (.62)	75.52 (.64)
DSST (% correct items) (*SD*)	67.30 (2.13)	43.61 (1.86)
MWT-B (% correct items) (*SD*)	21.26 (.91)	27.35 (.84)

DSST = Digit Symbol Substitution Test (adapted from Wechsler [47]); MWT-B = Multiple Choice Knowledge Test, Version B (adapted from Lehrl [48])

Participants performed two psychometric tests to assess components of fluid and crystallyzed intelligence. The Digit Symbol Substitution Test (DSST; adapted from Wechsler [47]) served as a measure of processing speed, and the Multiple-Choice Knowledge Test (MWT-B; adapted from Lehrl [48]) captured a person’s verbal knowledge. Younger adults performance was better than that of older adults in the DSST (*t*(44) = 8.39; *p* < .01, two-tailed). In contrast, the elderly were better in the MWT-B (*t*(44) = 4.90; *p* < .01, two-tailed).These findings match the idea of declining fluid and preserved crystallized intelligence with increasing age [49].

### Task, stimuli, and procedure

In the main experiment, participants completed a probabilistic learning task, in which they learned via feedback to press the accurate response button to an imperative stimulus [for similar learning tasks, see 10, 22]. The experimental stimuli were taken from the database of Snodgrass and Vanderwart [50]. A total of 50 stimuli were used, two of them from the fruit category for the practice session. Of the remaining stimulus material, eight stimuli each could be assigned to six different categories during the learning task (household items, tools, animals, furniture, toys, and clothing). The images were presented on a gray background in the middle of a 24-inch computer screen and scaled to a size of 98 x 140 pixels or 140 x 98 pixels. The screen resolution was 1024 x 768 pixels. As feedback stimuli, faces of four different persons were taken from the “FACES” database [51]. Due to the fact that both younger and older adults participated in the study, we chose older and younger, female and male persons as feedback faces to avoid age and gender biases in face processing. In the emotional condition, faces expressing the emotion “disgust” were chosen to indicate an incorrect answer and faces showing the emotion “joy” were chosen to signal a correct answer. Although prior research has used angry facial expressions as negative feedback, we decided to use disgusted faces because there is evidence that recognition of angry faces is particularly disrupted in older adults [for a meta-analytic review, see 52]. In the neutral feedback condition, neutral face stimuli of the same persons were used, i.e., their expression did not contain any emotion. In this condition, the background color of the feedback stimuli (yellow or blue) indicated whether the subject’s response was correct or incorrect. Feedback stimuli and background color in the neutral condition were counterbalanced over participants.

Overall, the experiment consisted of 960 trials, which were divided into six blocks each with 160 trials. Each trial started with a fixation cross, which was presented for 500 ms. The participants were then shown one of eight stimuli of the same category (e.g. furniture), which they could assign to one of two response buttons (the keys c and m on the keyboard) within 600–1500 ms. If participants exceeded the response time frame of 600–1500 ms, the message “too slow!” (German: “zu langsam!”) was displayed and the next trial followed. In order not to disadvantage the older subjects in the learning task, the response time that was available to the subjects was adjusted adaptively: The response time to the stimulus started at 1000 ms. After 19 trials the response time available was adjusted to 100 ms more or less, depending on the number of timeouts the subject caused. If the subject answered all 19 trials for all stimuli in the given time window, the response time was shortened to 100 ms. As soon as the participants exceeded the response time frame more than once, the response time was increased to 100 ms. Due to the adaptivity of the response time window, the minimum response time available to a subject was 600 ms and the maximum response time was 1500 ms. Additionally, after the subject responded, either a white frame representing the white key, or a black frame representing the black key appeared for 500 ms. The aim of presenting this frame was to visualize the given response, which might be especially helpful for older participants and thus reduce behavioral age differences in learning. For a period of 500 ms an empty screen followed before the feedback was presented. After assigning the stimuli to one of the buttons, the participants received feedback on their response, which was presented for 700 ms. In 90% of the cases the feedback was valid, i.e., the given feedback corresponded to the answer. In 10% of the cases the feedback was invalid, i.e., the feedback did not match the answer and the subjects received negative feedback after a correct answer and vice versa. This was done to avoid one-trial-learning. Please note that most probabilistic learning tasks use a probability ration of 80% of valid and 20% of invalid feedback. However, these studies also still find clear age differences between younger and older adults (e.g., Eppinger et al., 2008; Ferdinand, 2019). Because our impression from earlier studies was that older adults particularly struggle with the probabilistic nature of the feedback, we decided to use a “less probabilistic” feedback ratio of 90% to 10% to hopefully further reduce age differences in learning rates. Each stimulus was also linked to a specific feedback image of a person’s face and a feedback type (emotional vs. neutral). Thus, four stimuli in one learning block were followed by emotional feedback, and the remaining four were followed by neutral feedback. After the feedback presentation a blank screen followed (the inter-trial interval being 300 ms) and then the next trial started.

The probabilistic stimulus-response association task was explained to the participants in form of a cover story: They should imagine themselves as relocation helpers who were to load objects either in a black or white truck and indicate their decision by pressing a corresponding white or black response key (the keys c or m on the keyboard). They received feedback from their superiors who judged the assignment to be correct or incorrect. The probabilistic nature of the feedback was explained by saying that in very rare cases, these superiors could also be mistaken but that their feedback was reliable in most cases. Before each learning block, there was a reminder of what the superiors looked like and what their facial expressions (friendly, disgusted) or the coloring of the pictures (yellow, blue) stood for. Before the experiment started, the participants could familiarize themselves with the task in a practice session. During the learning block, eight stimuli of the same category were presented 20 times each in a random order. In a learning block, four stimuli were assigned to the white key (white truck) and four to the black key (black truck). After each block there was a short break, the length of which the subjects could determine themselves. In the following block, the category of learning stimuli changed and the learning task started again with new stimuli. The order in which the categories followed each other was balanced across participants. A learning block took about ten minutes and the duration of the experiment was about 60 minutes.

After the welcome and information on the general procedure, the participants completed a declaration of consent and a demographic questionnaire. Participants then performed a paper-and-pencil version of the DSST and a computerized version of the MWT-B. Afterwards they completed the handedness questionnaire by Oldfield [53] and the Interpersonal Reactivity Index (IRI, [54–55]), a questionnaire on empathy in specific domains. Finally, the participants filled in the Lüdenscheider activity questionnaire [56] as well as the Health Enhancement Lifestyle Profile-Screener (HELP, [57], translated into German), a questionnaire assessing lifestyle activities in several domains (results of these questionnaires will be reported elsewhere). After completing the questionnaires, participants were prepared for EEG recording and started the learning task. After completion, a recognition test for the learned stimuli (results will be reported elsewhere) and a short follow-up questionnaire (asking participants about their impressions of the emotional face stimuli, whether they had used strategies, and about comments on the experiment) was carried out. Finally, participants received their compensation and left.

### EEG recording and pre-processing

The EEG recording took place during the performance of the probabilistic learning task. The EEG was recorded from 58 active silver-silver chloride (Ag/AgCl) electrodes embedded in an elastic cap in the extended 10–20 system [58]. The electrode at position AFz served as a ground. The reference electrode was placed on the left mastoid, and an electrode was also attached to the right mastoid, which was used for referencing. For later correction of eye movements during the EEG recording, an electrooculogram was additionally recorded. For this purpose, an electrode was placed above and below the right eye and one electrode at the outer corners of the right and left eye. The impedances of the EEG and EOG electrodes were kept below 20 kΩ using electrolyte gel.

The EEG and EOG signal was filtered online through a low pass filter (250 Hz). The analogue-to-digital conversion was performed at a sampling rate of 500 Hz. Before analyzing the data, the EEG signal was filtered offline using a bandpass filter of 0.01 to 30 Hz. Horizontal and vertical eye movements were corrected by a linear regression approach [59]. All other artifacts in the EEG data were rejected prior to averaging the data if the standard deviation of the amplitude in a 200 ms interval was above 30 μV in the ocular electrodes or 20 μV in the representative electrode Cz. In addition, a visual inspection of the EEG signal took place in the relevant time windows, in which trials with artifacts were rejected and not included in data averaging. This way, in the group of younger participants 17.0% (SEM = .14) of trials were rejected and in the group of older adults 14.3% (SEM = .17) of trials were rejected because of artifacts. A t-test for independent samples showed no difference between the groups (*t*(44) = 41.64, *p* < .01, two-tailed).The preparation of the EEG data was carried out by EEProbe (ANT software).

### Statistical analyses

For the analysis of the behavioral data, accuracy and reaction times were measured in the probabilistic learning task. Any responses that did not occur within the adaptive presentation duration of each stimulus were not considered for data analysis. In order to be able to present and evaluate the learning process of the subjects, the individual learning blocks were divided into quarters (160 trials each). The mean of the reaction times and accuracy were first calculated for each subject per learning quarter in the individual learning blocks and then averaged across all six learning blocks. This procedure was done for both feedback types (emotional, neutral). The data were then analyzed with a mixed ANOVA and included the between-subject factor age group (older, younger adults) and the within-subject factors feedback type (emotional, neutral) and learning quarter (quarters 1, 2, 3, 4). Whenever the factor learning quarter was significant, it was followed up by pairwise comparisons (quarter 1 vs. 2, 2 vs. 3, and 3 vs. 4) in order to limit pairwise comparisons to a minimum. Also, because we did not have any specific hypotheses about the slope of the learning curves, these comparisons were Bonferroni-corrected.

The analysis of the EEG data was based on the ERP waveforms linked to the feedback presentation. From the ongoing EEG, time windows of 900 ms were chosen, which included a 200 ms baseline before stimulus onset (-200 ms to 700 ms). To capture the reward prediction error, the FRN was measured in a peak-to-peak fashion between the positivity in a time window from 180 ms to 240 ms and the following negativity in a time window from 240 ms to 330 ms after feedback presentation [cf. 10–11, 60]. The P3b was defined as the mean amplitude in the time window between 400 ms and 600 ms after feedback presentation. The chosen time window of 400–600 ms for the relevant feedback conditions (emotional, neutral / negative, positive) was based on previous studies and on the inspection of the mean P3b-amplitudes for both age groups. Since the P3b is particularly pronounced on posterior electrodes and shows age-related changes in the distribution to anterior scalp regions, the midline electrodes Fz, Cz, and Pz were used for the analysis of P3b-amplitude. To test the postulated effects, a mixed ANOVA was calculated for the analysis of the ERP data. This included the between subject factor age group (older, younger adults) and the within subject factors feedback type (emotional, neutral), valence (negative, positive) and electrode position (AnteriorPosterior; Fz, Cz, Pz).

If sphericity was violated, Greenhouse-Geisser correction was applied to both behavioral and ERP data and corrected p-values along with the corresponding Greenhouse-Geisser ε value and uncorrected degrees of freedom are reported. In case of post-hoc testing, the adjustment of the alpha level for multiple comparisons was done using Bonferroni correction. The significance level was set to α = .05 in all analyzes. Statistical analysis was performed using IBM SPS Statistics Version 23.

## Results

### Accuracy

**Fig 1:**
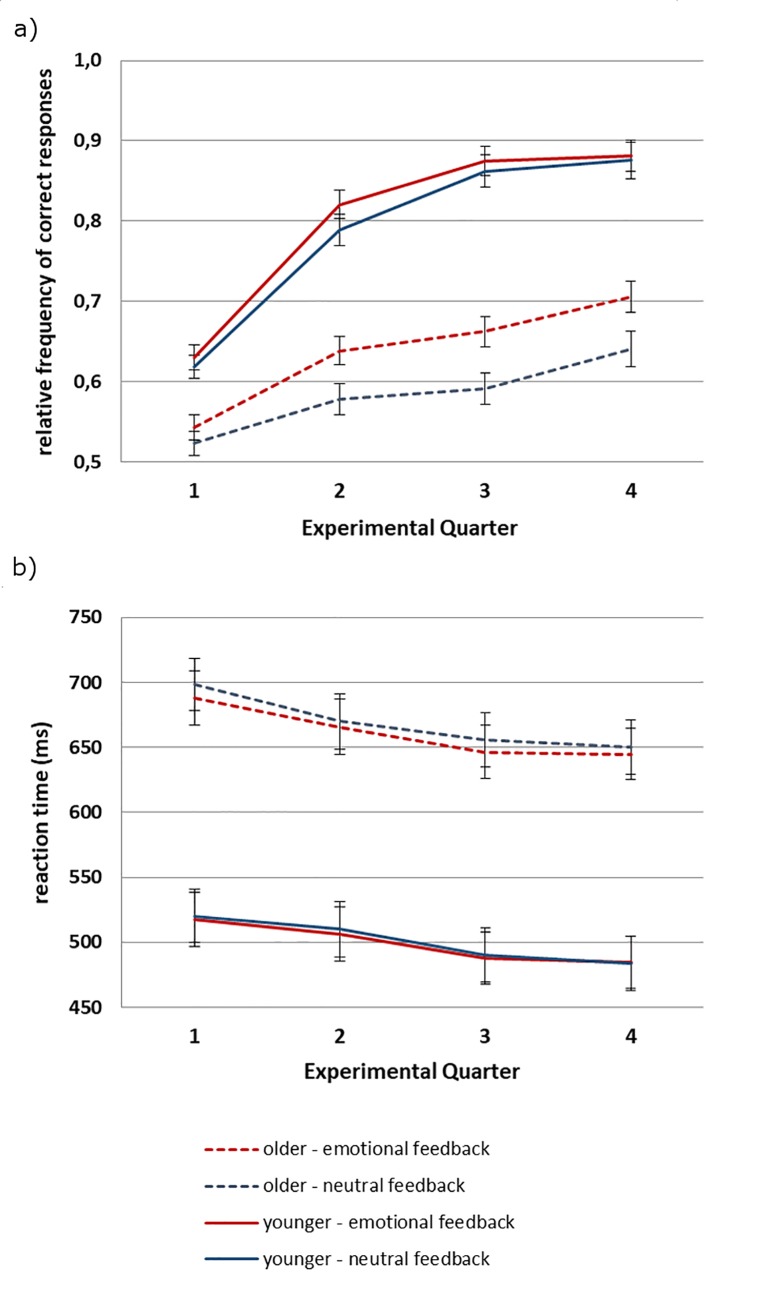
a) Accuracy and b) reaction time data for younger and older participants in the emotional and the neutral condition of the probabilistic learning task (whiskers denote standard errors of the mean).

Participants accuracy in the probabilistic learning task (see Fig 1A) was analyzed by means of a repeated-measures ANOVA with the between-subjects factor Age (younger, older), and the within-subjects factors Feedback Condition (emotional, neutral) and Experimental Quarter (1, 2, 3, 4). This ANOVA resulted in a main effect of Age (*F*(1,44) = 93.85, *p* < .01, *η*_*p*_*^2^* = .68), indicating lower accuracies for older (mean = .61, SEM = .01) than younger adults (mean = .79, SEM = .01). Additionally, main effects for Feedback Condition (*F*(1,44) = 12.94, *p* < .01, *η*_*p*_*^2^* = .23), and Experimental Quarter (*F*(3,132) = 157.84, *p* < .01, *η*_*p*_*^2^* = .78), and interactions between Age and Condition (*F*(1,44) = 4.08, *p* < .05, *η*_*p*_*^2^* = .09) and Age and Quarter (*F*(3,132 = 21.74, *p* < .01, *η*_*p*_*^2^* = .33) were found.

To find the locus of these interactions, separate ANOVAs with the factors Feedback Condition and Experimental Quarter were calculated for the two age groups. For younger adults, this ANOVA yielded a main effect of Quarter (*F*(3,66) = 212.20, *p* < .01, *η*_*p*_*^2^* = .91), reflecting increasing accuracy from Quarter 1 to 3 (Quarter 1 vs. 2: *F*(1,22) = 156.89, *p* < .01, *η*_*p*_^2^ = .88; Quarter 2 vs. 3: *F*(1,22) = 89.02, *p* < .01, *η*_*p*_*^2^* = .80), while no effect of Feedback Condition was found (p = .17). For older adults, the ANOVA also showed a main effect of Experimental Quarter (*F*(3,66) = 26.76, *p* < .01, *η*_*p*_*^2^* = .55), with increasing accuracy from Quarter 1 to 2 (*F*(1,22) = 19.09, *p* < .01, *η*_*p*_*^2^* = .47) and from Quarter 3 to 4 (*F*(1,22) = 29.76, *p* < .01, *η*_*p*_*^2^* = .58). Moreover, it resulted in a main effect of Feedback Condition (*F*(1,22) = 11.52, *p* < .01, *η*_*p*_*^2^* = .34), with better accuracy in the emotional condition for older adults only.

### Reaction times

In analogy to the accuracy data, reaction times (see Fig 1B) were analyzed be means of a repeated-measures ANOVA with the between-subjects factor Age (younger, older), and the within-subjects factors Condition (emotional, neutral) and Experimental Quarter (1, 2, 3, 4). This ANOVA yielded a main effect of Age (*F*(1,44) = 25.66, *p* < .01, *η*_*p*_*^2^* = .37), indicating faster reaction times for younger (mean = 504ms, SEM = 20) than older adults (mean = 650ms, SEM = 20), and a main effect of Experimental Quarter (*F*(3,132) = 58.23, *p* < .01, *η*_*p*_*^2^* = .57), reflecting decreasing reaction times over the course of the task (Quarter 1 vs. 2: *F*(1,44) = 45.48, *p* < .01, *η*_*p*_*^2^* = .51; Quarter 2 vs. 3: *F*(1,44) = 32.57, *p* < .01, *η*_*p*_*^2^* = .43; Quarter 3 vs. 4: *F*(1,44) = 4.39, *p* < .05, *η*_*p*_*^2^* = .09). Moreover, a main effect of Condition was significant (*F*(1,44) = 8.97, *p* < .01, *η*_*p*_*^2^* = .17), demonstrating faster reaction times in the emotional than the neutral condition.

Because we had the hypothesis that older adults should profit more from the emotional feedback condition than younger adults, we additionally conducted separate ANOVAs for the two age groups. These ANOVAs yielded a main effect of Experimental Quarter for younger (*F*(3,66) = 47.26, *p* < .01, *η*_*p*_*^2^* = .68) as well as older adults (*F*(3,66) = 23.63, *p* < .01, *η*_*p*_*^2^* = .52). However, a main effect of Condition indicating faster reaction times for emotional than neutral trials was significant only for the older (*F*(1,22) = 6.14, *p* < .05, *η*_*p*_*^2^* = .22) but not the younger adults (*p* = .10).

### Peak-to-peak FRN

**Fig 2:**
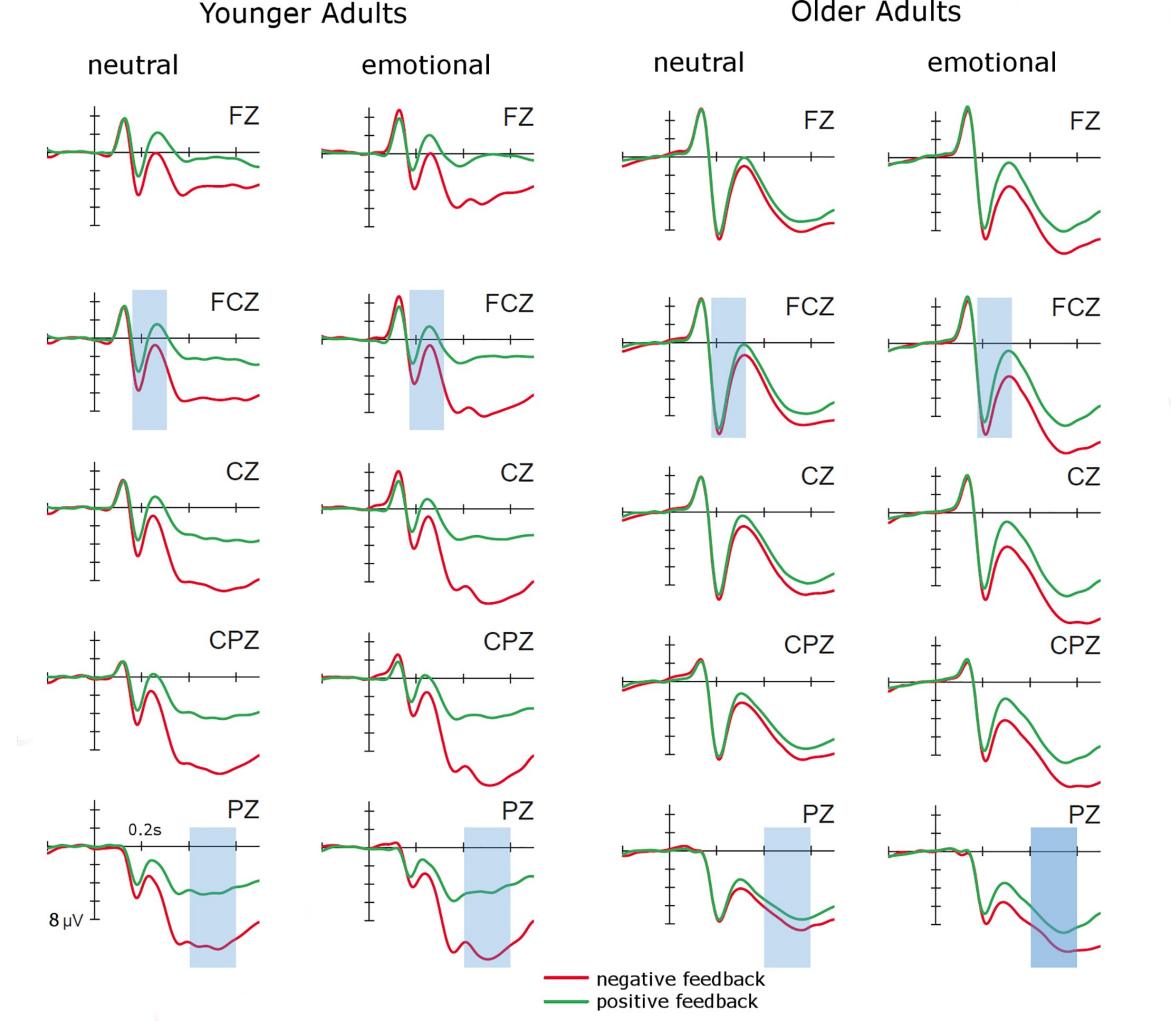
Feedback-locked ERPs for younger and older participants in the emotional and the neutral condition of the probabilistic learning task at electrodes Fz, FCz, Cz, CPz, and Pz.

**Fig 3:**
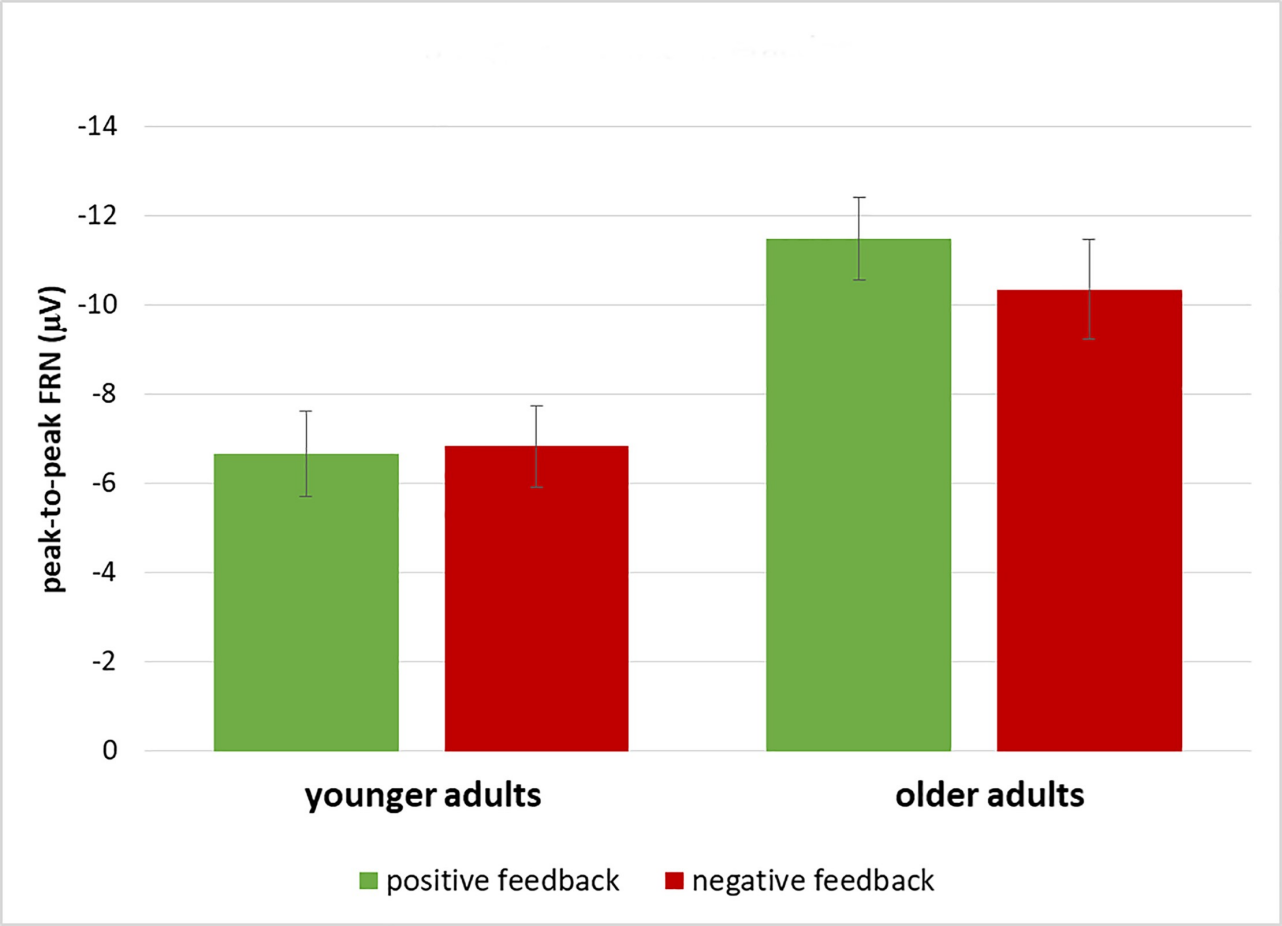
Illustration of the interaction between age and feedback valence in the peak-to-peak FRN (whiskers denote standard errors of the mean).

The peak-to-peak FRN at FCz (see Fig 2) was analyzed in an ANOVA with the between-subjects factor Age (younger, older), and the within-subjects factors Condition (emotional, neutral), and Feedback Valence (positive, negative). It resulted in a main effect of Age (*F*(1,44) = 10.51, *p* < .01, *η*_*p*_*^2^* = .19), reflecting larger peak-to-peak FRNs for older than younger adults, and a main effect of Condition (*F*(1,44) = 12.78, *p* < .01, *η*_*p*_*^2^* = .23), reflecting larger peak-to-peak FRNs for the neutral than the emotional condition. Additionally, the ANOVA yielded an interaction between Age and Feedback Valence (*F*(1,44) = 5.55, *p* < .05, *η*_*p*_*^2^* = .11), which was due to a larger FRN after positive than negative feedback for older (*F*(1,22) = 15.79, *p* < .01, *η*_*p*_*^2^* = .42) but not for younger adults (*p* = .74; see Fig 3).

### P3b

**Fig 4:**
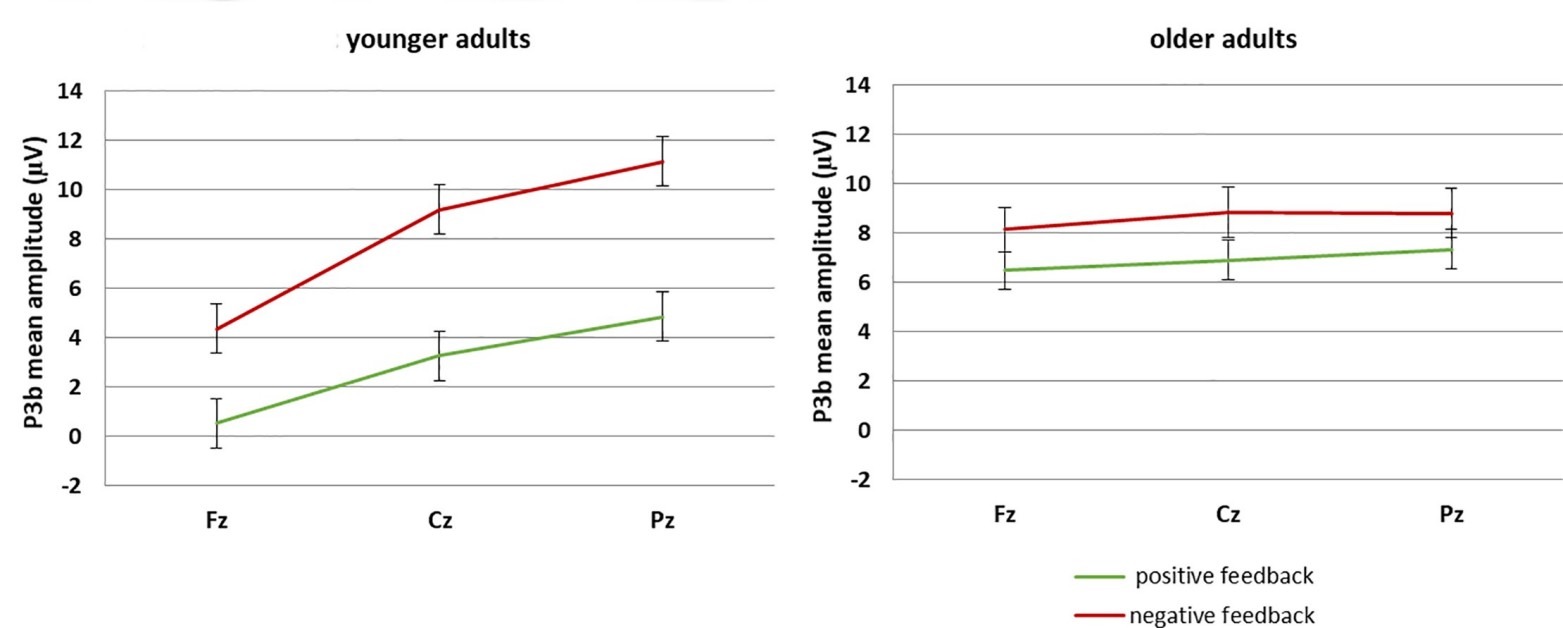
Illustration of the interaction between feedback valence and anterior/posterior in younger adults’ P3b mean amplitudes (whiskers denote standard errors of the mean). This effect was absent in the older adults’ P3b data.

An ANOVA with the between-subjects factor Age (younger, older), and the within-subjects factors Feedback Condition (emotional, neutral), Feedback Valence (positive, negative), and Anterior/Posterior (Fz, Cz, Pz) was conducted to analyze P3b mean amplitude (see Fig 4). This ANOVA resulted in main effects of Feedback Condition (*F*(1,44) = 27.60, *p* < .01, *η*_*p*_*^2^* = .39), Feedback Valence (*F*(1,44) = 106.69, *p* < .01, *η*_*p*_*^2^* = .71), and Anterior/Posterior (*F*(2,88) = 36.97, *p* < .01, *η*_*p*_*^2^* = .46), and interactions between Age and Feedback Condition (*F*(1,44) = 11.74, *p* < .01, *η*_*p*_*^2^* = .21), Age and Feedback Valence (*F*(1,44) = 29.01, *p* < .01, *η*_*p*_*^2^* = .40), Age and Anterior/Posterior (*F*(2,88) = 21.11, *p* < .01, *η*_*p*_*^2^* = .32), Feedback Condition and Feedback Valence (*F*(1,44) = 7.86, *p* < .01, *η*_*p*_*^2^* = .15), Feedback Valence and Anterior/Posterior (*F*(2,88) = 19.06, *p* < .01, *η*_*p*_*^2^* = .30), and Age, Feedback Valence, and Anterior/Posterior (*F*(2,88) = 19.45, *p* < .01, *η*_*p*_*^2^* = .31). To explain these multiple effects from the overall ANOVA, matching our hypotheses separate ANOVAs with the factors Feedback Condition, Feedback Valence, and Anterior/Posterior were calculated for the two age groups.

For the younger adults, this ANOVA resulted in a main effect of Feedback Valence (*F*(1,22) = 103.10, *p* < .01, *η*_*p*_*^2^* = .82), a main effect of Anterior/Posterior (*F*(2,44) = 47.61, *p* < .01, *η*_*p*_*^2^* = .68) and an interaction between these two factors (*F*(2,44) = 25.50, *p* < .01, *η*_*p*_*^2^* = .54). As can be seen in Fig 4, these effects mean that the P3b had a posterior distribution for positive (Fz vs. Cz: *F*(1,22) = 46.86, *p* < .01, *η*_*p*_*^2^* = .68; Cz vs. Pz: *F*(2,44) = 19.70, *p* < .01, *η*_*p*_*^2^* = .47) and negative (Fz vs. Cz: *F*(1,22) = 60.35, *p* < .01, *η*_*p*_*^2^* = .73; Cz vs. Pz: *F*(2,44) = 18.84, *p* < .01, *η*_*p*_*^2^* = .46) feedback. Additionally, positive feedback elicited a smaller P3b than negative feedback at all three electrode locations (Fz: *F*(1,22) = 69.42, *p* < .01, *η*_*p*_*^2^* = .76; Cz: *F*(1,22) = 86.70, *p* < .01, *η*_*p*_*^2^* = .80; Pz: *F*(1,22) = 111.30, *p* < .01, *η*_*p*_*^2^* = .84). The interaction between Feedback Valence and Anterior/Posterior in the younger adults’ P3b data was probably due to larger differences between positive and negative feedback at posterior electrodes, as can be inferred from the effect sizes.

**Fig 5:**
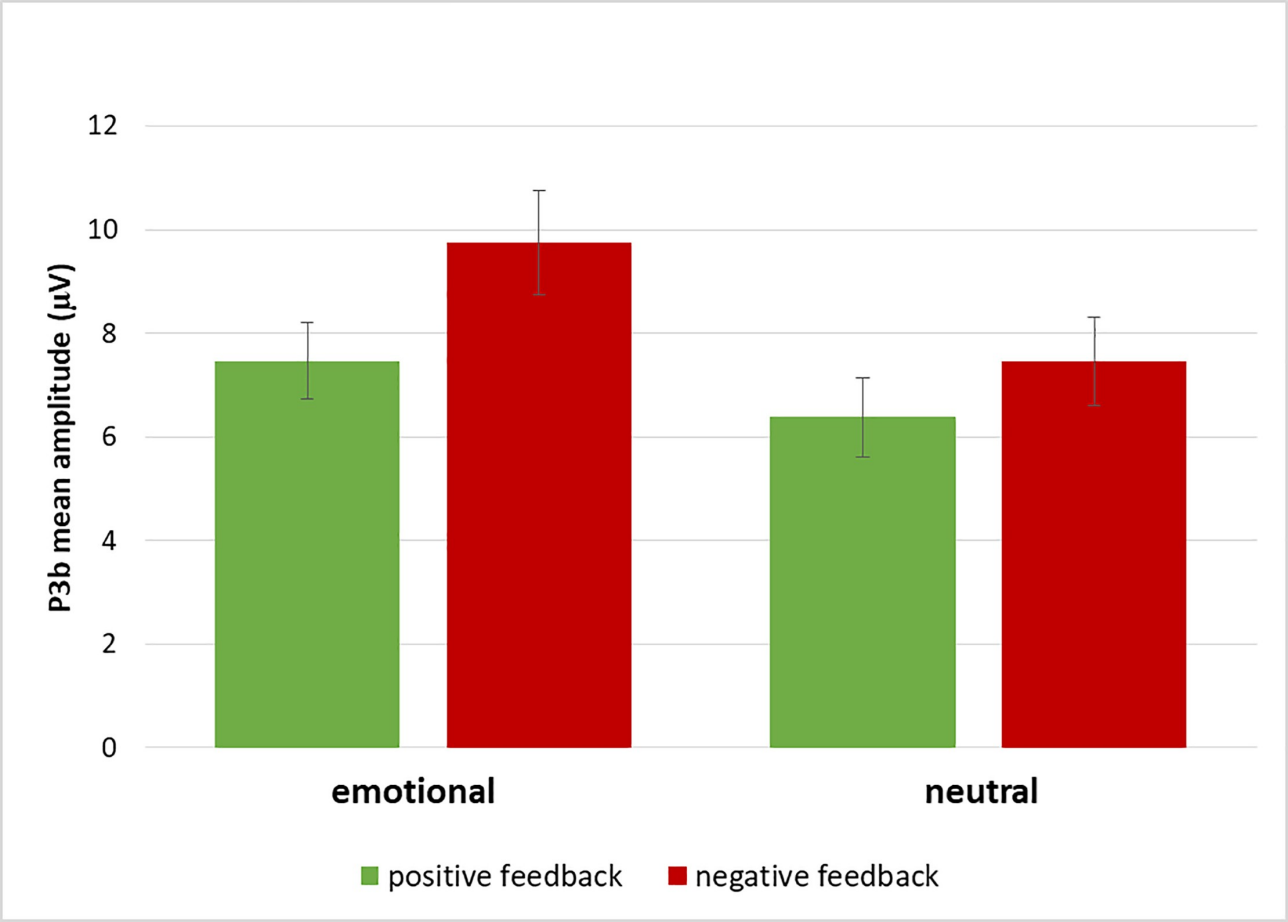
Illustration of the main effects of feedback condition and feedback valence in older adults’ P3b mean amplitudes (whiskers denote standard errors of the mean).

For the older adults, this ANOVA resulted in a main effect of Feedback Condition (*F*(1,22) = 40.38, *p* < .01, *η*_*p*_*^2^* = .65), indicating a larger P3b after emotional than neutral feedback, and a main effect of Feedback Valence (*F*(1,22) = 15.23, *p* < .01, *η*_*p*_*^2^* = .41), demonstrating a larger P3b after negative than positive feedback (see Fig 5). There was no main effect nor interactions including the factor Anterior/Posterior in older adults P3b data (all p-values >.15; see also Fig 4).

To sum up, the P3b results show a clear parietal distribution in younger adults with larger amplitudes for negative than positive feedback. Such a parietal distribution was not found for older adults (see Fig 4). Instead, for older adults, larger P3b amplitudes were found for negative and for emotional feedback (see Fig 5).

### Post-hoc analyses

**Fig 6:**
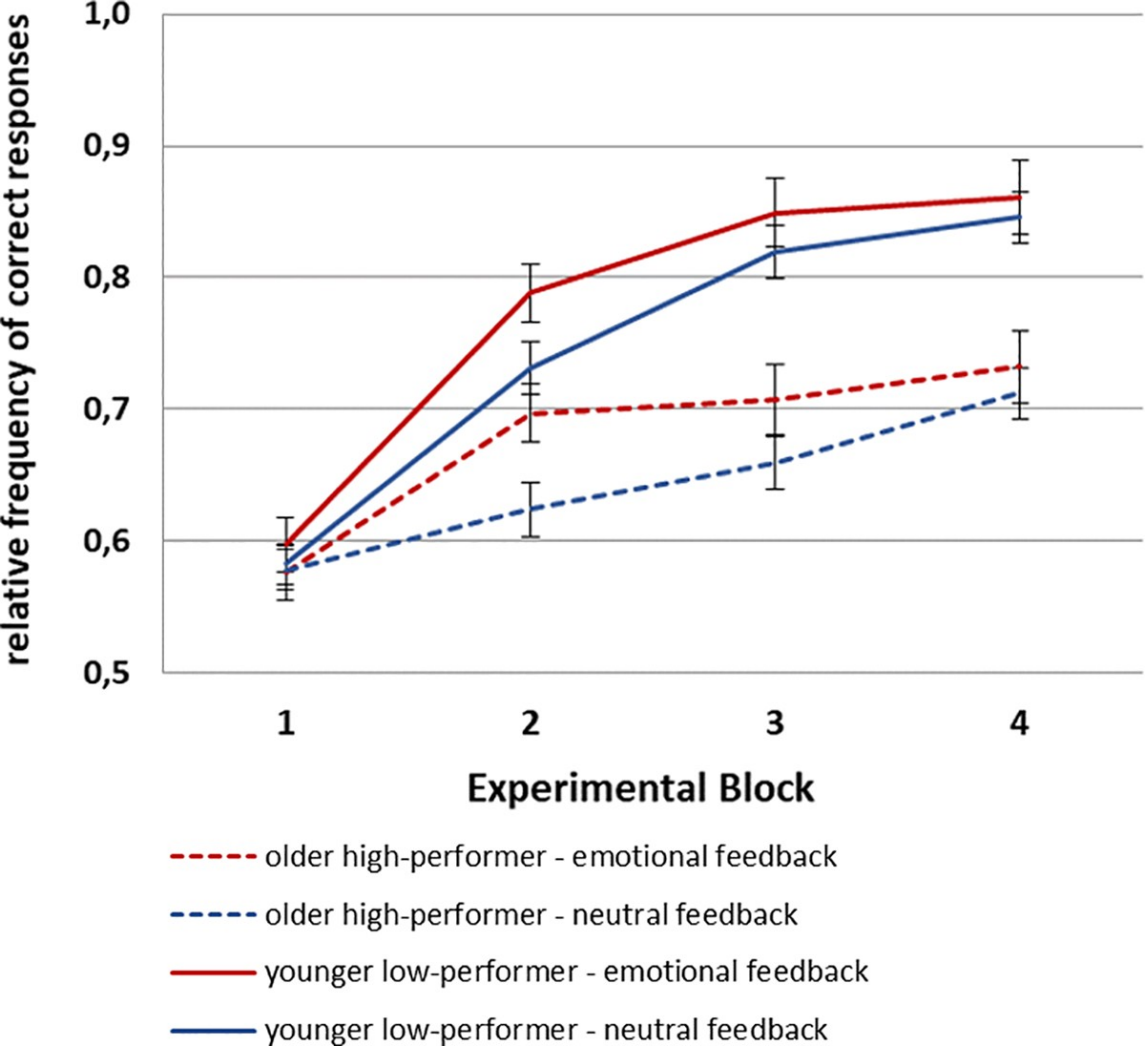
Accuracy data for younger poor-performers and older high-performers in the emotional and the neutral conditions of the probabilistic learning task.

Because older adults’ differences in P3b amplitude between the neutral and the emotional feedback condition might be due to the differences in their performance between the two conditions, we conducted a median split on the accuracy data (from neutral trials) and compared twelve older high-performers with twelve younger poor-performers (median = .80 for younger and median = .60 for older adults). The ANOVA on the accuracy data (see Fig 6) with the factors Age, Feedback Condition, and Experimental Quarter still found significant effects of Age (*F*(1,22) = 30.52, *p* < .01, *η*_*p*_*^2^* = .58), Feedback Condition (*F*(1,22) = 5.15, *p* < .05, *η*_*p*_*^2^* = .19), Experimental Quarter (*F*(3,66) = 87.10, *p* < .01, *η*_*p*_*^2^* = .80) and interactions between Age and Quarter (*F*(3,66) = 9.96, *p* < .01, *η*_*p*_*^2^* = .31) and Feedback Condition and Quarter (*F*(3,66) = 3.10, *p* < .05, *η*_*p*_*^2^* = .12). However, when separate ANOVAs were calculated for each age group, there was only an effect of Experimental Quarter left for both younger (*F*(3,33) = 84.93, *p* < .01, *η*_*p*_*^2^* = .89) and older (*F*(3,33) = 18.40, *p* < .01, *η*_*p*_*^2^* = .63) adults and effects of Feedback Condition were lacking (all *p*-values >.12). This means that at least within each age group, performance in the two feedback conditions is comparable.

In the next step, we checked whether the P3b results changed when performance differences between the two feedback conditions were absent within each age group. For this reason, the ANOVA with the factors Age, Feedback Condition, Feedback Valence, and Anterior/Posterior was repeated. It resulted in main effects of Age (F(1,22) = 5.62, p < .05, η_p_^2^ = .20), Feedback Condition (F(1,22) = 10.51, p < .01, η_p_^2^ = .32), Feedback Valence (F(1,22) = 76.65, p < .01, η_p_^2^ = .78), and Anterior/Posterior (F(2,44) = 15.46, p < .01, η_p_^2^ = .41), and interactions between Age and Feedback Condition (F(1,22) = 6.09, p < .01, η_p_^2^ = .22), Age and Feedback Valence (F(1,22) = 8.01, p < .05, η_p_^2^ = .27), Age and Anterior/Posterior (F(2,44) = 12.83, p < .01, η_p_^2^ = .37), Feedback Valence and Anterior/Posterior (F(2,44) = 12.65, p < .01, η_p_^2^ = .37), and Age, Feedback Valence, and Anterior/Posterior (F(2,44) = 12.57, p < .01, ηp^2^ = .36). The interaction between Feedback Condition and Feedback Valence was marginally significant (F(1,22) = 3.18, p = .09, η_p_^2^ = .13).

Separate ANOVAs with the factors Feedback Condition, Feedback Valence, and Anterior/Posterior for each age group showed main effects of Feedback Valence (F(1,11) = 44.52, p < .01, η_p_^2^ = .80) and Anterior/Posterior (F(2,22) = 36.40, p < .01, η_p_^2^ = .77) and an interaction between these two factors (F(2,22) = 18.08, p < .01, η_p_^2^ = .62) for younger adults. Again, although the P3b had a posterior distribution for positive (Fz vs. Cz: *F*(1,11) = 17.35, *p* < .01, *η*_*p*_*^2^* = .61; Cz vs. Pz: *F*(1,11) = 10.64, *p* < .01, *η*_*p*_*^2^* = .49) and negative (Fz vs. Cz: *F*(1,11) = 55.16, *p* < .01, *η*_*p*_*^2^* = .83; Cz vs. Pz: *F*(1,11) = 13.86, *p* < .01, *η*_*p*_*^2^* = .56) feedback and positive feedback elicited a smaller P3b than negative feedback at all three electrode locations (Fz: *F*(1,11) = 34.32, *p* < .01, *η*_*p*_*^2^* = .76; Cz: *F*(1,11) = 40.32, *p* < .01, *η*_*p*_*^2^* = .79; Pz: *F*(1,11) = 48.69, *p* < .01, *η*_*p*_*^2^* = .82), the interaction was probably due to larger differences between positive and negative feedback at posterior electrodes, as can be inferred from the effect sizes. For the older adults, this ANOVA resulted in a main effect of Feedback Condition (*F*(1,11) = 16.08, *p* < .01, *η*_*p*_*^2^* = .59), indicating a larger P3b after emotional than neutral feedback, and a main effect of Feedback Valence (*F*(1,11) = 35.62, *p* < .01, *η*_*p*_*^2^* = .76), demonstrating a larger P3b after negative than positive feedback. Thus, the pattern of results in P3b mean amplitude did not change with performance.

## Discussion

The goal of the present study was to explore whether emotional feedback ameliorates age-related impairments in feedback-induced learning and feedback processing. To this end, younger and older participants conducted a probabilistic reinforcement learning task in which the accurate responses had to be learned via feedback. In emotional trials, feedback stimuli consisted of faces with smiling and disgusted expressions, and in a non-emotional condition, positive and negative feedback was indicated by the background color of faces with neutral expressions.

The behavioral data indicate that although older adults show slower reaction times in general, younger as well as older adults learned to assign the correct response keys to the different stimuli in the probabilistic learning task. This was reflected in increasing accuracies and decreasing reaction times over the course of the experiment. Still, younger adults showed better and faster learning, as can be inferred from the increasing accuracy from the first to the third quarter of the experiment, but no further improvement in the fourth quarter, i.e., younger adults seemed to have reached their learning plateau. In contrast, older adults still showed improvements in performance from the third to the fourth quarter of the experiment and also reached lower levels of learning. Most importantly, both reaction times and accuracy data revealed that older adults learned better in trials with emotional than neutral feedback. This effect of emotion was not present in younger adults. This is in line with the findings by Gorlick and colleagues [45], who found that emotional feedback ameliorated age-related impairments in learning, but had no effect on younger adults learning performance. However, the results are in contrast to the behavioral findings of Hurlemann and colleagues [39] who found better performance in conditions with emotional than abstract feedback in an item-category learning task with young adult males. Moreover, electrophysiological studies suggest that emotional feedback is processed more strongly in younger adults, although the findings on whether this also shows in the performance data are mixed [e.g., 40–42]. A possible explanation of these mixed findings could be, that the use of emotional feedback is dependent on the task difficulty, i.e., younger adults do profit from emotional feedback as the older adults do, but this benefit only shows in more demanding tasks. In this sense, the lack of emotional benefit in the learning rates of younger adults might be due to a ceiling effect. Whether younger adults would have profited from emotional feedback, if the learning task had been more difficult for them, is an open question for future research.

When analyzing the peak-to-peak FRN, we found that it was larger for neutral than for emotional feedback stimuli. This was rather unexpected, because we had hypothesized that emotional feedback would lead to increased feedback processing even in this fast initial phase of feedback processing. However, the peak-to-peak FRN is known to reflect expectancy violations, which can then result in learning processes. Thus, faces with neutral expressions seem to elicit larger expectancy violations than emotional faces. In hindsight, one could speculate that this is due to the fact that neutral faces that still convey negative or positive feedback (via a different aspect than facial expression) might present a rather artificial and unusual stimulus in a social situation where feedback from another person is expected (as was suggested by the instruction of the task). Nevertheless, we decided to use these neutral expressions because they have an equal perceptual complexity than the emotional face stimuli and feedback complexity is known to influence the FRN [41, 61]. To explicitly explore this possibility, however, further research is needed. Another unexpected finding was that older adults had a larger peak-to-peak FRN than younger adults. Usually, the opposite pattern is found [21–27]. We can only speculate that this is related to the processing of faces which might be especially important for older adults because—according to the socio-emotional selectivity theory [35, 62]—they invest more effort in emotional well-being and thus emotional expectancy violations might be of greater importance. Here, too, further research is needed comparing face feedback to other kinds of emotional feedback to explore this possibility. A third finding was a larger peak-to-peak FRN after positive than negative feedback in older but not younger adults. Because the peak-to-peak FRN reflects a violation of expectancies, this probably reflects the fact that positive feedback still presents an expectancy violation for older adults when this is no longer the case for younger adults, which is in line with older adults’ slower and worse learning [for a similar argument, see 10, 14, 22]. Interestingly, this pattern of FRN findings does not correspond to the learning pattern in the behavioral data. Otherwise, every signaling of an expectancy violation should result in a learning improvement, i.e., we should have found better learning in the neutral condition and better learning in older adults. Instead, our FRN findings show that expectancy violations can be violations that are helpful for learning to accomplish the task at hand, but they can also be driven by other unexpected aspects which are not so helpful to solve the present task–like the fact that a stimulus itself is rather unusual (e.g., the neutral faces in this task). This suggests that the cognitive mechanism reflected in the FRN is a very basic detection mechanism for expectancy violations, probably functioning as an alerting signal, which then needs to be followed by more elaborated cognitive mechanisms that are more specifically tuned to the goal of the present task.

In a next step, we examined the later and more elaborated stage of feedback processing which is reflected in the P3b and related to the updating of the current working memory contents [17–18]. We replicated a typical age-effect in P3b scalp distribution. While the P3b was clearly parietally distributed in the young adults, for the older adults, it had a broader scalp distribution which extended into central and frontal areas. This distribution pattern is commonly thought of as a mechanism compensating for working memory deficits in older adults. It indicates that it is necessary for the elderly to additionally recruit frontal brain regions to be able to properly process the feedback stimulus and update working memory [10; 34; 63–65]. Second, we found that for younger as well as older adults, the P3b was larger after negative as compared to positive feedback. This result matches learning studies using similar tasks [10, 66] and probably shows that negative feedback is perceived as being more relevant for the current learning task than positive feedback. While positive feedback in most cases only represents an affirmation that the correct reaction is already known and applied, negative feedback is a sign that the reaction was inadequate and behavior needs to be adapted. To achieve this, more updating of the internal models of the environment (i.e., the present learning task) needs to take place [17–18]. Only for older adults, we found an additional effect of emotional condition, i.e., they had a larger P3b after emotional than non-emotional feedback. Thus, only older adults engaged in working memory updating more strongly after they had received emotional feedback. This is consistent with their behavioral data, where they reached the best learning performance in trials with emotional feedback. Hence, more working memory updating in this condition might have led to better learning performance. Thus, this updating mechanism (reflected in the P3b) is much more specific to the learning task at hand than the mere detection of an expectancy violation of any kind (indexed by the peak-to-peak FRN). In line with this view, the pattern of P3b results matches the pattern of the behavioral learning effects.

### Limitations

One limitation of the reported data could be that the P3b is related to surprising, unexpected events, i.e., the less frequent the feedback, the larger the P3b. In a learning task, this means that the better the learning performance, the less frequent the negative feedback. In other words, the larger P3b in emotional trials in older adults could be an effect of negative feedback frequency instead of an effect of emotion. However, there are two arguments that speak against this frequency interpretation. First, we obtained a main effect of emotional condition, i.e., negative as well as positive emotional feedback elicited a larger P3b than neutral (negative and positive) feedback. Second, to show that the present results are independent of learning performance (and thus feedback frequency), we compared older high-performers and younger low-performers. Although these results have to be interpreted with caution because of the small sample size, this comparison demonstrated that the pattern of results did not change: Even for older high-performers, whose learning performance did not differ between the emotional and the non-emotional feedback condition, the P3b was still larger for emotional than non-emotional feedback. On the other hand, the effect of emotional feedback was still absent for younger low-performers. This supports the idea that the emotional effects described above were not due to feedback frequencies, but genuine emotional effects.

Another limitation was, that the emotional face feedback had several unexpected effects on early feedback processing as reflected in the FRN. We speculated above that this might be related to the effects of emotional faces on older adults. Nevertheless, this suggestion needs to be examined by future research. To this end, other types of emotional feedback should be compared to emotional face feedback in younger, but especially in older adults. Additionally, a neutral feedback condition that does not include faces at all, but which is of similar perceptual complexity (e.g., scrambled faces), would be a helpful comparison condition. These procedures could help clarify the open questions concerning the fast and initial feedback processing as reflected in the FRN, which is less related to effortful, controlled processing and much more affected by perceptual stimulus properties.

## Conclusion

Our results demonstrate that emotional feedback can diminish age-related impairments in learning and feedback processing in older adults. To our knowledge, this study is the first to shed light on some of the underlying neuronal mechanisms by analyzing the brain activity related to feedback processing. We showed that emotional face feedback led to better learning performance in older adults as compared with non-emotional feedback, probably by strengthening the feedback’s relevance and thus working memory updating. Whether a similar strengthening effect of emotional feedback might also be helpful for younger adults under more demanding task conditions remains an open question for future research.
